# IgG subclass levels in referred hemochromatosis probands with *HFE* p.C282Y/p.C282Y

**DOI:** 10.1371/journal.pone.0302817

**Published:** 2024-05-14

**Authors:** James C. Barton, J. Clayborn Barton, Luigi F. Bertoli, Ronald T. Acton

**Affiliations:** 1 Department of Medicine, University of Alabama at Birmingham, Birmingham, Alabama, United States of America; 2 Southern Iron Disorders Center, Birmingham, Alabama, United States of America; 3 Department of Medicine, Brookwood Baptist Medical Center, Birmingham, Alabama, United States of America; 4 Department of Microbiology, University of Alabama at Birmingham, Birmingham, Alabama, United States of America; The University of Hong Kong, HONG KONG

## Abstract

**Background:**

IgG subclass levels in hemochromatosis are incompletely characterized.

**Methods:**

We characterized IgG subclass levels of referred hemochromatosis probands with *HFE* p.C282Y/p.C282Y (rs1800562) and human leukocyte antigen (HLA)-A and -B typing/haplotyping and compared them with IgG subclass levels of eight published cohorts of adults unselected for hemochromatosis.

**Results:**

There were 157 probands (82 men, 75 women; mean age 49±13 y). Median serum ferritin, mean body mass index (BMI), median IgG4, and median phlebotomy units to achieve iron depletion were significantly higher in men. Diabetes, cirrhosis, and HLA-A*03,-B*44, -A*03,B*07, and -A*01,B*08 prevalences and median absolute lymphocyte counts in men and women did not differ significantly. Mean IgG subclass levels [95% confidence interval] were: IgG1 5.31 g/L [3.04, 9.89]; IgG2 3.56 g/L [1.29, 5.75]; IgG3 0.61 g/L [0.17, 1.40]; and IgG4 0.26 g/L [<0.01, 1.25]. Relative IgG subclasses were 54.5%, 36.6%, 6.3%, and 2.7%, respectively. Median IgG4 was higher in men than women (0.34 g/L [0.01, 1.33] vs. 0.19 g/L [<0.01, 0.75], respectively; p = 0.0006). A correlation matrix with Bonferroni correction revealed the following positive correlations: IgG1 vs. IgG3 (p<0.01); IgG2 vs. IgG3 (p<0.05); and IgG2 vs. IgG4 (p<0.05). There was also a positive correlation of IgG4 vs. male sex (p<0.01). Mean IgG1 was lower and mean IgG2 was higher in probands than seven of eight published adult cohorts unselected for hemochromatosis diagnoses.

**Conclusions:**

Mean IgG subclass levels of hemochromatosis probands were 5.31, 3.56, 0.61, and 0.26 g/L, respectively. Median IgG4 was higher in men than women. There were positive associations of IgG subclass levels. Mean IgG1 may be lower and mean IgG2 may be higher in hemochromatosis probands than adults unselected for hemochromatosis.

## Introduction

Hemochromatosis in whites of western European descent is associated with homozygosity for *HFE* p.C282Y (rs1800562), a common missense allele of the homeostatic iron regulator (chromosome 6p22.2) in linkage disequilibrium with human leukocyte antigen (HLA)-A*03 [1, 2]. HFE, a non-classical class I major histocompatibility complex (MHC) protein, is an upstream regulator of hepcidin and thus of iron homeostasis [3]. Laboratory phenotypes of many adults at diagnosis of hemochromatosis and p.C282Y/p.C282Y include elevated levels of transferrin saturation (TS) and serum ferritin (SF) [4]. Adults with p.C282Y/p.C282Y have increased risks of developing iron overload. Severe iron overload occurs predominantly in men [4, 5]. Non-*HFE* heritable and environmental variables modify iron loading in adults with hemochromatosis [2, 4, 6, 7]. Some adults with p.C282Y/p.C282Y also have hemochromatosis arthropathy, diabetes mellitus, hypogonadotropic hypogonadism, hepatic cirrhosis, or cardiomyopathy [4].

The prevalence of hemochromatosis TS/SF phenotypes and *HFE* p.C282Y homozygosity was significantly greater in 240 index patients with common variable immunodeficiency or immunoglobulin (Ig) G subclass deficiency than in 318 unrelated control subjects [8]. In a subsequent report, subnormal levels of IgG subclass 1 (IgG1), IgG3, or IgG1/IgG3 based on 1996 consensus guidelines [9] were common in 51 referred hemochromatosis probands with p.C282Y homozygosity [10] and there was concordance of Ig and hemochromatosis TS/SF phenotypes in probands and their HLA-identical siblings [10]. Thus, it was postulated that a putative allele on chromosome 6p haplotypes bearing either p.C282Y or HLA-A*03 influences IgG subclass levels [10].

Aims of this study are 1) to characterize serum IgG subclass levels at diagnosis in a replication cohort of 157 referred hemochromatosis probands with *HFE* p.C282Y homozygosity and HLA-A and -B typing/haplotyping, 2) to investigate laboratory and clinical associations with IgG subclass levels of this cohort, and 3) to compare mean and relative levels of IgG subclasses levels of this cohort with those of eight previously published adult cohorts unselected for hemochromatosis diagnoses. We discuss the present observations in the context of variables that influence IgG subclass levels of adults with and without diagnoses of hemochromatosis and p.C282Y homozygosity.

## Methods

### Ethics statement

This retrospective work was performed according to the principles of the Declaration of Helsinki [43]. Performance of this study was approved by Western Institutional Review Board, Inc. (submission 2539985–44189619). Western Institutional Review Board, Inc. waived the need for obtaining informed consent from participants in this study under United States Department of Health and Human Services, Office for Human Research Participants, regulation 45 CFR 46.101(b)(4). Obtaining informed consent was not required and thus was not obtained because this study involved retrospective chart review and analyses of observations recorded in routine medical care. Data analyzed in this study were not anonymized before the investigators accessed them because data were compiled from proband charts in an Alabama tertiary hematology center wherein JaCB and LFB diagnosed and treated all probands, consistent with Western Institutional Review Board, Inc. approval of this study. JaCB, JClB, and LFB had access to information that could identify individual probands during and after data collection. Data were accessed for research purposes during the interval 30 December 2018–3 June 2020. All data in this report are displayed in a manner that maintains proband anonymity in both the present results and corresponding dataset.

### Subjects included

We retrospectively compiled data of all consecutive self-identified non-Hispanic whites aged ≥18 y referred to an Alabama tertiary hematology center during the study interval 1 January 2007–30 October 2018 for evaluation and management of hemochromatosis who met the following criteria: a) had *HFE* p.C282Y/p.C282Y, b) had no known non-hemochromatosis iron disorder, c) underwent measurement of IgG subclasses at diagnosis, d) underwent HLA-A and -B typing, e) achieved iron depletion with therapeutic phlebotomy, as appropriate, and f) were the first in their respective families to be diagnosed to have hemochromatosis (probands).

Medical histories were taken from probands and records of referring physicians. Referring physicians diagnosed and treated probands with diabetes. Physicians in the present hematology center evaluated probands for cirrhosis, as appropriate. All probands underwent medication review, physical examination, laboratory testing, imaging procedures, and evaluation of liver and other conditions, as indicated, before therapeutic phlebotomy was initiated [11].

### Subjects excluded

We excluded probands with the following: a) hyperferritinemia, hemochromatosis, or *HFE* p.C282Y/p.C282Y diagnosed as a consequence of family or population screening, b) diagnosis of a primary or secondary hematologic disorder, c) volunteer whole-blood donation >two units in the year before hemochromatosis diagnosis, d) bariatric operation [12], e) viral hepatitis B or C, f) liver transplant, g) diagnosis of malignancy, h) anti-cancer therapy, i) non-iron-related chronic inflammatory condition, j) self-reported pregnancy, k) monoclonal or polyclonal gammopathy, or l) previous diagnosis of primary antibody deficiency.

We also excluded probands who reported current or recent therapy with any of the following medications previously associated with altered IgG or IgG subclass levels: a) hydroxychloroquine [13]; corticosteroids [14, 15]; captopril, carbamazepine, chloroquine, diphenylhydantoin, fenclofenac, gold compounds, hydantoin, levamisole, penicillamine, sulfasalazine, valproic acid, or zonisamide [16]; oxcarbazapine [17]; leflunomide [18], methotrexate [19], rituximab [20]; intravenous or subcutaneous polyclonal IgG [21]; or intravenous or subcutaneous monoclonal IgG.

### Laboratory

Blood specimens were collected during mornings without regard to fasting. Complete blood counts were measured ≤1 h after diagnostic phlebotomy using an automated hematology analyzer (Cell-Dyn^®^ Model 610, Model 1700, Model 1800, or Emerald (Abbott Laboratories, Chicago, IL, USA)). Reference range for absolute lymphocyte count (ALC) was the same for each analyzer (0.6–4.1 x 10^6^/L). TS and SF were measured using standard methods (Laboratory Corporation of America, Burlington, NC, USA). We defined these TS and SF levels to be elevated: TS >50% (men) and TS >45% (women); and SF >300 μg/L (men) and SF >200 μg/L (women) [22, 23].

Serum IgG and IgG subclass levels at diagnosis were measured using rate nephelometry (Laboratory Corporation of America, Burlington, NC, USA). The following previously suggested reference limits were based on 1996 consensus guidelines [9]: IgG 7.00–16.00 g/L (700–1600 mg/dL); IgG1 4.22–12.92 g/L (422–1292 mg/dL); IgG2 1.17–7.47 g/L (117–747 mg/dL); IgG3 0.41–1.29 g/L (41–129 mg/dL); and IgG4 0.01–2.91 g/L (1–291 mg/dL) (Laboratory Corporation of America, Burlington, NC, USA).

*HFE* genotyping was performed as previously described [24]. We determined HLA-A and -B types and haplotypes as previously described [24, 25]. We studied HLA-A*03, the optimal marker for the hemochromatosis ancestral haplotype [24], and HLA-A*03, B*07, the most common HLA-A and -B haplotype in p.C282Y homozygotes [25]. We also studied two other markers, prevalences of which are increased in adults with subnormal IgG subclass levels and frequent/severe respiratory tract infection: HLA-B*44 [15, 26, 27] and HLA-A*01, B*08 [15, 26]. Positivity for HLA types and haplotypes was defined as either homozygosity or heterozygosity.

### Iron removed to achieve iron depletion

Iron depletion therapy, defined as the periodic removal of blood to eliminate storage iron, was performed in all probands with elevated SF levels (>300 μg/L (men) and >200 μg/L (women)) as described elsewhere [28]. We defined 450–500 mL of blood removed at a single phlebotomy session as one unit. Iron depletion therapy was complete when SF was ≤20 μg/L [28]. We defined the number of units of blood removed to achieve iron depletion in probands without elevated SF as zero.

### IgG subclasses in previously reported adult cohorts

We performed computerized and manual searches to identify representative reports of IgG subclass levels of cohorts of >50 adults. We selected cohorts for tabulation based on study population, methodology used to quantify IgG subclass levels, and computation and display of mean IgG subclass levels.

### Statistics

We evaluated the medical records of 169 referred probands and excluded 12 (7.1%; three with viral hepatitis B or C, three with polyclonal gammopathy, two with monoclonal gammopathy, and four with insufficient data). The dataset for the present analyses includes observations in the remaining 157 hemochromatosis probands and is available in Figshare repository [29]. TS measures before phlebotomy treatment commenced were unavailable in six of 157 probands (3.8%; three men, three women) [29]. Seven of 157 probands (4.5%; six men, one woman) did not complete phlebotomy to achieve iron depletion [29]. Thus, TS and therapeutic phlebotomy data are reported herein for 151 probands and 150 probands, respectively [29].

We display age, TS, and SF data as integers, body mass index (BMI), ALC, and phlebotomy units to achieve iron depletion data with one significant decimal place, and IgG subclass data with two or three significant figures. IgG4 levels reported as <0.01 g/L were imputed as 0.005 g/L. We excluded total IgG measures from most analyses because IgG levels of adults are determined predominantly by IgG1 and IgG2 levels [30, 31]. We compared 15 characteristics of the previous cohort of 51 hemochromatosis probands [10] with those of the present replication cohort of 157 hemochromatosis probands.

Frequency distributions of IgG subclass levels, displayed as smoothed curves, depict percentages of 157 probands as functions of ten subgroups of the corresponding IgG subclass levels. Error bars represent 95% confidence intervals (CI) for the proportions of probands in each of the ten subgroups. We defined IgG subclass levels <2 SD below the corresponding means as subnormal.

Kolmogorov-Smirnov testing demonstrated that age and BMI data did not differ significantly from those which are normally distributed. We displayed these data as means ± 1 standard deviation (SD) and compared them using Student’s t test for unpaired data (two-tailed). TS, SF, ALC, and phlebotomy units to achieve iron depletion data differed significantly from those which are normally distributed. We displayed these data as medians (range) and compared them using Mann-Whitney U test (two-tailed). In other analyses, we used *ln* transformations to adjust IgG subclass data to Gaussian distributions. Means of IgG subclass levels, 95% CI, and percentiles (2.5, 5, 10, 25, 50, 75, 90, 95, 97.5) were determined from the means and SDs of the natural logarithm (*ln*) data and transformed as anti-*ln* values to the original scale for presentation. Categorical variables were compared using Fisher’s exact test (two-tailed).

To identify relationships of IgG subclass values with other variables, we computed a correlation matrix with Bonferroni corrections on the variables sex, age, TS, SF, BMI, IgG subclass levels, and positivity for A*03 and B*44.

We used Excel^®^ 2000 (Microsoft Corp., Redmond, WA, USA) and GraphPad Prism 8^®^ (2018; GraphPad Software, San Diego, CA, USA). We defined p <0.05 to be significant, although we used Bonferroni corrections to reduce the likelihood of type I errors in multiple univariate and bivariate comparisons.

## Results

### Validation of a replicate hemochromatosis proband cohort

We compared 15 characteristics of a 2003 hemochromatosis proband cohort (n = 51) [10] with those of the present probands (n = 157) (Table 1). Comparisons revealed no significant differences between these cohorts after Bonferroni correction, demonstrating that the present cohort of 157 probands is a replicate of the 2003 cohort.

**Table 1:** Two cohorts of referred hemochromatosis probands with *HFE* p.C282Y/p.C282Y^a^.

Characteristic	2003 cohort (n = 51)^b^	Present cohort (n = 157)	Value of p^c^
Mean age, y ± SD	48 ± 13	49 ± 13	0.4255
Men, % (n)	52.9 (27)	52.2 (82)	~1.0000
Diabetes, % (n)	9.8 (5)	16.6 (26)	0.3646
Cirrhosis, % (n)	5.9 (3)	8.3 (13)	0.7658
Median TS, % (range)^d^	89 (38, 100)	83 (41, 100)	0.0308
Median SF, μg/L (range)	619 (247, 5000)	706 (28, 5630)	0.9124
IgG1 <4.21 g/L, % (n)^e^	23.5 (12)	23.6 (37)	~1.0000
IgG2 <1.17 g/L, % (n)	0 (0)	0.6 (1)	~1.0000
IgG3 <0.41 g/L, % (n)^e^	29.4 (15)	27.4 (43)	~1.0000
IgG4 <0.01 g/L, % (n)	3.9 (2)	2.5 (4)	0.6365
HLA-A*03 positivity, % (n)^f^	76.5 (39)	70.7 (111)	0.4763
HLA-B*44 positivity, % (n)^f^	23.5 (12)	24.2 (38)	0.8259
HLA-A*01, B*08 positivity, % (n)^f^	9.8 (5)	11.5 (18)	~1.0000
HLA-A*03, B*07 positivity, % (n)^f^	45.1 (23)	42.0 (66)	0.7459
Median phlebotomy units to achieve iron depletion (range)	20 (5, 150)	15 (0, 150)^g^	0.0143

^a^*HFE*, homeostatic iron regulator; HLA, human leukocyte antigen; Ig, immunoglobulin; IgG, immunoglobulin G; SD, standard deviation; SF, serum ferritin; TS, transferrin saturation. No proband had hypogonadotropic hypogonadism or cardiomyopathy attributed to iron overload. All characteristics were determined at diagnosis except phlebotomy units to achieve iron depletion.

^b^The 2003 cohort is described in detail elsewhere [10].

^c^Significant value of p after Bonferroni correction = 0.0033.

^d^TS data are reported for 150 probands.

^e^Six probands (11.8%) from the 2003 cohort and 15 probands (9.6%) in the present cohort had both IgG1 <4.21 g/L and IgG3 <0.41 g/L at diagnosis (p = 0.6032).

^f^ Heterozygosity or homozygosity.

^g^Phlebotomy units data represent observations in 76 men and 74 women in the present cohort.

### Characteristics of 157 referred hemochromatosis probands

There were 82 men (52.2%) and 75 women (47.8%) (Table 2). TS was elevated in 75 men (91.5%) and 66 women (88.0%) (p = 0.5995). SF was elevated in 72 men (91.5%) and 64 women (85.3%) (p = 0.8150). After Bonferroni correction, median SF, mean BMI, mean IgG4, and median phlebotomy units to achieve iron depletion were significantly higher in men than women (Table 2).

**Table 2:** Referred probands with hemochromatosis and *HFE* p.C282Y/p.C282Y^a^.

Characteristic	Men (n = 82)	Women (n = 75)	Value of p^b^
Mean age at diagnosis, y ± SD	47 ± 13	50 ± 13	0.0728
Hemochromatosis arthropathy, % (n)	22.0 (18)	18.7 (14)	0.6932
Diabetes, % (n)	19.5 (16)	13.3 (10)	0.3907
Cirrhosis, % (n)	13.4 (11)	2.7 (2)	0.0189
Mean BMI, kg/m^2^ ± SD	29.1 ± 5.2	26.0 ± 5.5	0.0002
Median TS, % (range)^c^	88 (41, 100)	81 (42, 100)	0.0214
Median SF, μg/L (range)	957 (28, 5630)	511 (32, 5427)	<0.0001
Median ALC x 10^6^/L (range)	2.1 (0.8, 4.8)	2.1 (1.0, 4.2)	0.6745
Mean IgG1, g/L [95% CI]^d^^,^ ^e^	5.45 [2.76, 9.79]	5.26 [3.02, 11.3]	0.9463
Mean IgG2, g/L [95% CI]	3.69 [1.53, 7.19]	3.40 [1.16, 6.06]	0.1344
Mean IgG3, g/L [95% CI]^d^	0.61 [0.16, 1.43]	0.60 [0.17, 1.33]	0.7176
Mean IgG4, g/L [95% CI]	0.34 [0.01, 1.33]	0.19 [<0.01, 0.75]	0.0006
HLA-A*03 positivity, % (n)^f^	78.0 (64)	62.7 (47)	0.0372
HLA-B*44 positivity, % (n)^f^	19.5 (16)	29.3 (22)	0.1920
HLA-A*01, B*08 positivity, % (n)^f^	11.0 (9)	12.0 (9)	~1.0000
HLA-A*03, B*07 positivity, % (n)^f^	46.3 (38)	38.7 (29)	0.3387
Median phlebotomy units to achieve iron depletion (range)^g^	18 (0, 150)	12 (0,75)	<0.0001

^a^*HFE*, homeostatic iron regulator; HLA, human leukocyte antigen; Ig, immunoglobulin; IgG, immunoglobulin G; SD, standard deviation; SF, serum ferritin; TS, transferrin saturation. No proband had hypogonadotropic hypogonadism or cardiomyopathy attributed to iron overload. All characteristics were determined at diagnosis except phlebotomy units to achieve iron depletion.

^b^Significant value of p after Bonferroni correction = 0.0029.

^c^TS data represent observations in 79 men and 72 women.

^d^Ten men (12.2%) and five women (6.7%) had both IgG1 <4.21 g/L and IgG3 <0.41 g/L at diagnosis (p = 0.2852).

^e^These values represent means of natural logarithm (*ln*)-transformed values [95% CI].

^f^Heterozygosity or homozygosity.

^g^Phlebotomy units data represent observations in 76 men and 74 women.

### Frequency distributions of IgG subclass levels

**Fig 1:**
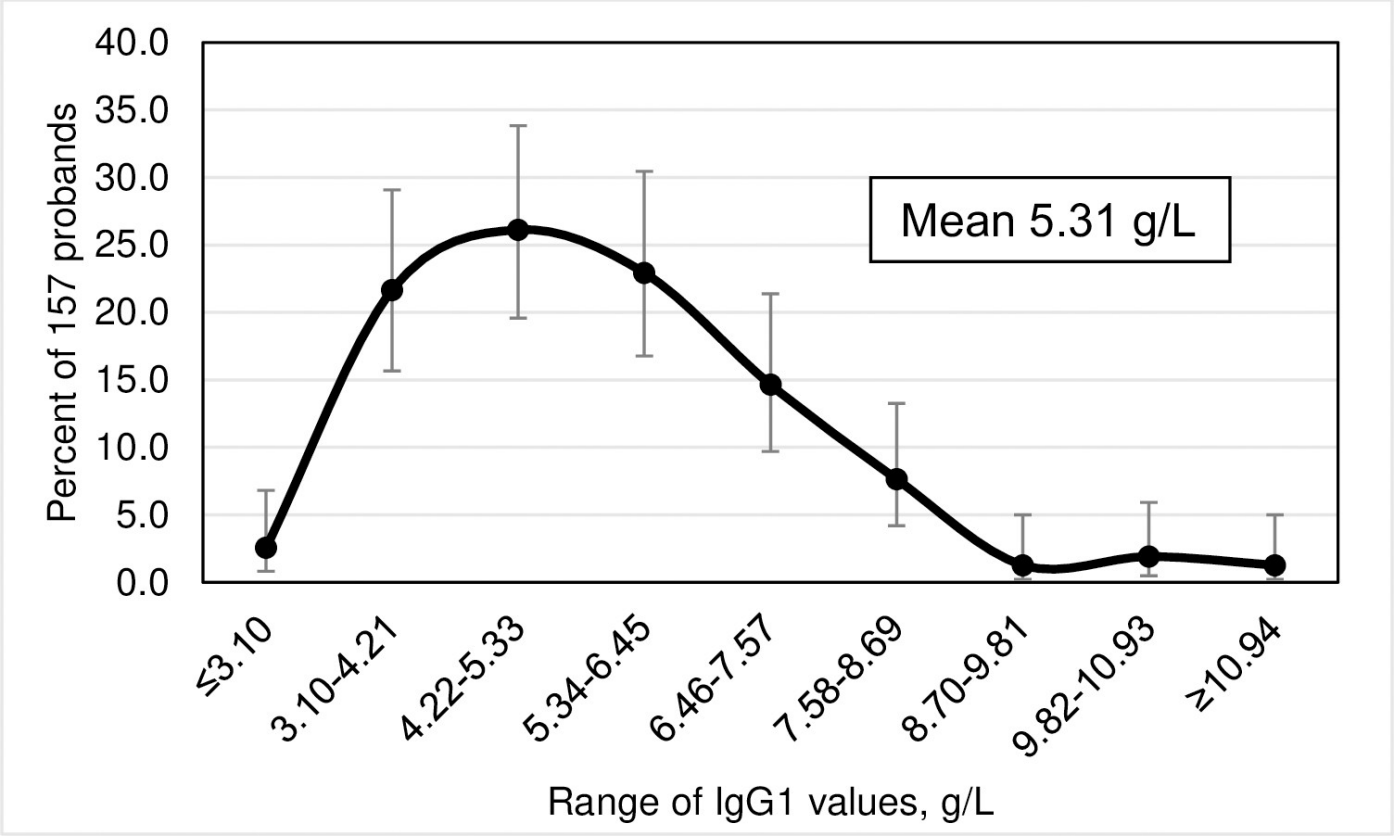
Smoothed frequency distribution of serum IgG1 subclass levels of 157 hemochromatosis probands with *HFE* p.C282Y homozygosity. Error bars represent 95% confidence intervals of proband percentages with continuity corrections.

**Fig 2:**
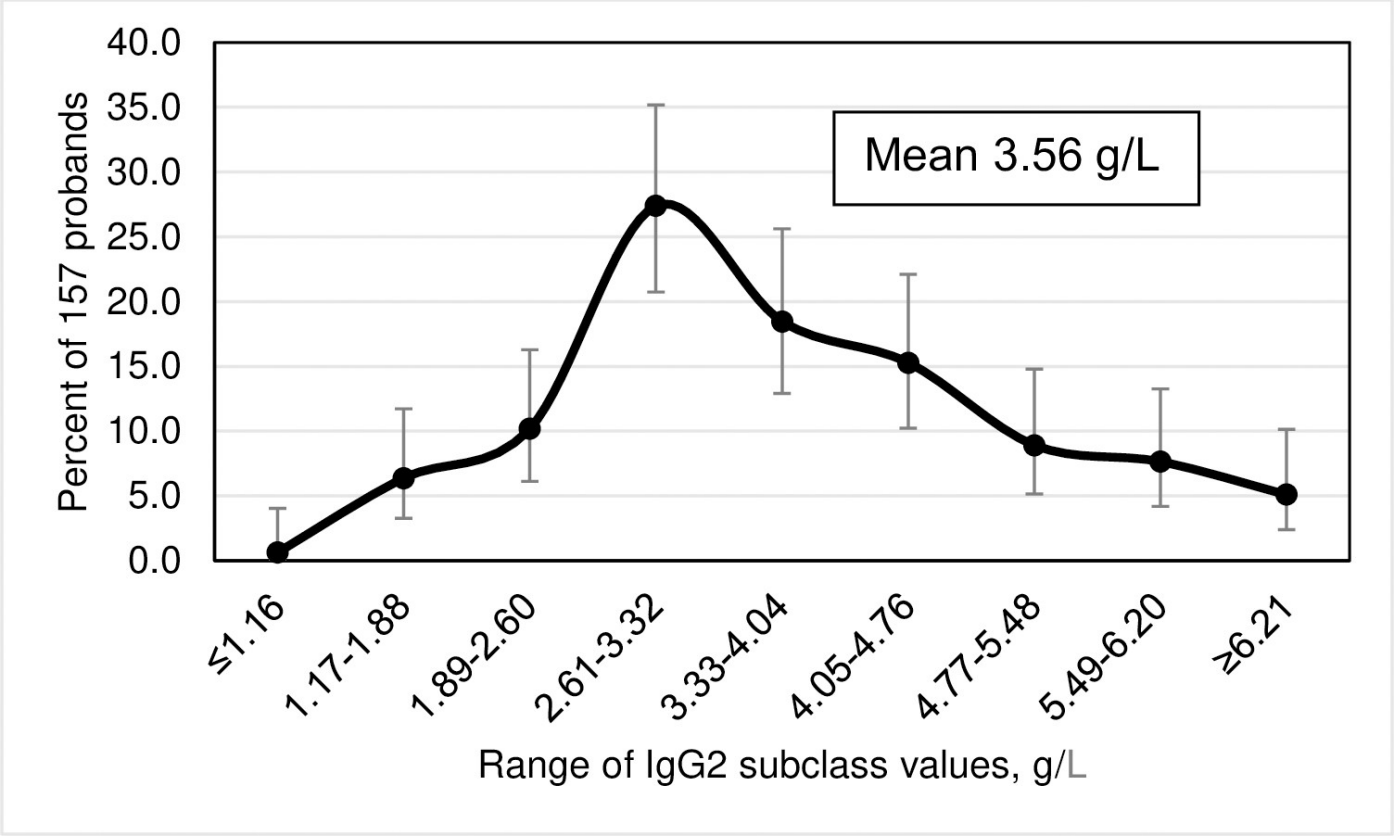
Smoothed frequency distribution of serum IgG2 subclass levels of 157 hemochromatosis probands with *HFE* p.C282Y homozygosity. Error bars represent 95% confidence intervals of proband percentages with continuity corrections.

**Fig 3:**
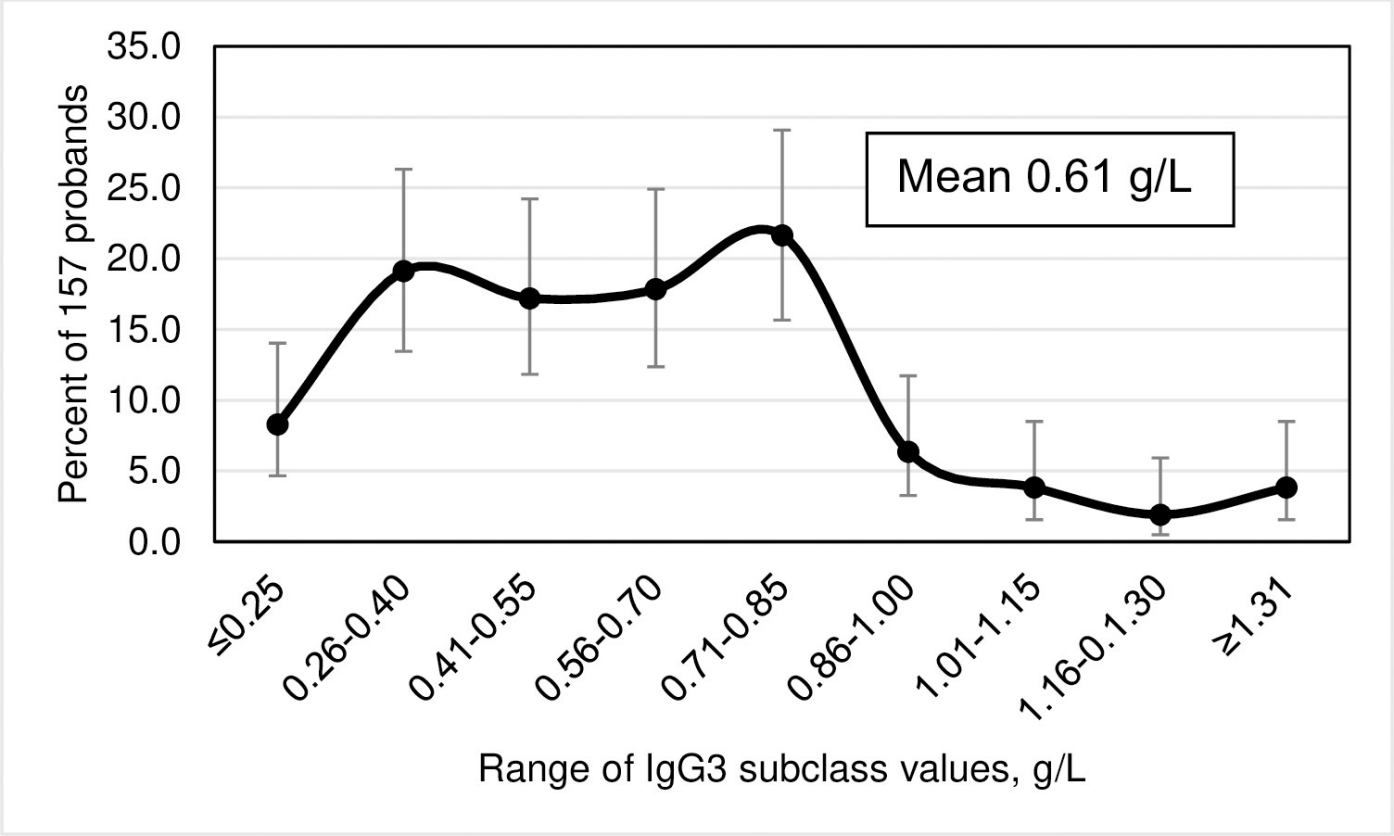
Smoothed frequency distribution of serum IgG3 subclass levels of 157 hemochromatosis probands with *HFE* p.C282Y homozygosity. Error bars represent 95% confidence intervals of proband percentages with continuity corrections.

**Fig 4:**
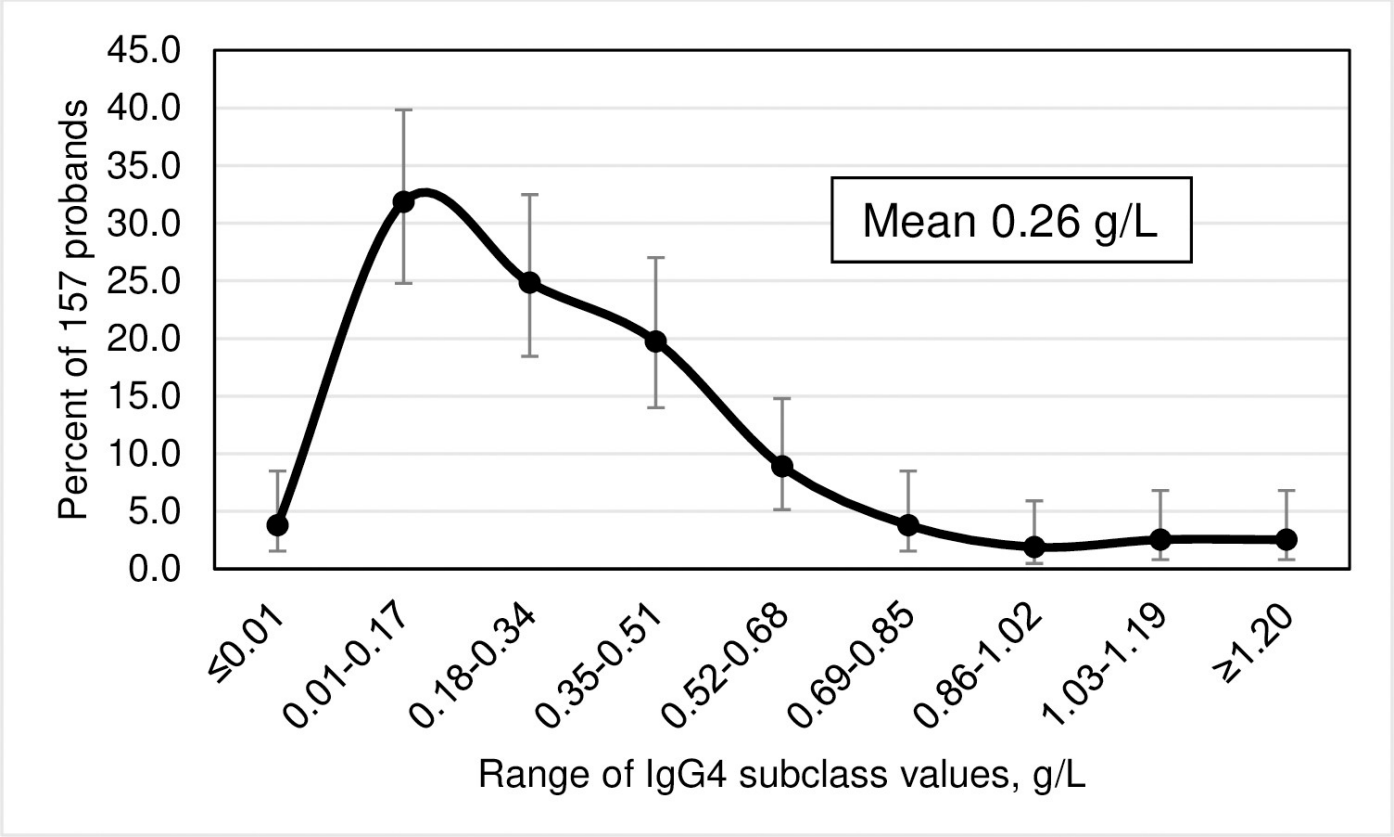
Smoothed frequency distribution of serum IgG4 subclass levels of 157 hemochromatosis probands with *HFE* p.C282Y homozygosity. Error bars represent 95% confidence intervals of proband percentages with continuity corrections.

The highest prevalence of IgG1 levels occurred in the range 4.22–5.33 g/L (Fig 1). The highest prevalence of IgG2 levels occurred in the range 2.61–3.32 g/L (Fig 2). The highest prevalence of IgG3 levels occurred in the range 0.71–0.85 g/L (Fig 3). The highest prevalence of IgG4 levels occurred in the range 0.01–0.17 g/L (Fig 4). Frequency distributions of all IgG subclass levels were right skewed. Ratios of the highest: lowest levels of respective IgG subclass levels were the following: IgG1 = 4.9: 1; IgG2 = 7.5: 1; IgG3 = 11.2: 1; and IgG4 = 282.1: 1. Pearson’s correlation of *ln* (IgG1+IgG2+IgG3+IgG4) vs. *ln* total IgG was strong (r = 0.9274; p <0.0001).

### Percentiles of IgG subclass levels

The respective percentile values of IgG1, IgG2, and IgG3 levels were similar in men and women. All percentile values of IgG4 levels were lower in women than men (Table 3).

**Table 3:** Percentiles of IgG subclass levels in referred hemochromatosis probands with *HFE* p.C282Y/p.C282Y.

IgG subclass	Percentile	2.5	5.0	10.0	25.0	50.0	75.0	90.0	95.0	97.5
IgG1, g/L	Men (n = 82)	2.76	3.42	3.76	4.23	5.45	6.33	7.92	8.69	9.79
	Women (n = 75)	3.02	3.17	3.63	4.33	5.26	6.99	7.84	9.00	11.3
IgG2, g/L	Men (n = 82)	1.53	1.61	2.17	2.86	3.69	4.61	6.04	6.98	7.19
	Women (n = 75)	1.16	1.29	2.11	2.73	3.40	4.41	5.31	5.75	6.06
IgG3, g/L	Men (n = 82)	0.16	0.22	0.26	0.36	0.61	0.83	1.06	1.38	1.43
	Women (n = 75)	0.17	0.22	0.27	0.38	0.60	0.76	0.90	1.05	1.33
IgG4, g/L	Men (n = 82)	0.01	0.06	0.09	0.16	0.34	0.52	1.00	1.24	1.33
	Women (n = 75)	<0.01	0.01	0.02	0.10	0.19	0.36	0.56	0.60	0.75

### Correlations of IgG subclass levels

We computed a correlation matrix (with Bonferroni corrections) on the variables sex, age, TS, SF, BMI, IgG subclass levels, and positivity for A*03 and B*44. This revealed the following positive correlations: IgG1 vs. IgG3 (p<0.01); IgG2 vs. IgG3 (p <0.05); and IgG2 vs. IgG4 (p <0.05). There was also a positive correlation of IgG4 vs. male sex (p <0.01).

### IgG subclass levels of adults with and without hemochromatosis diagnoses

Mean IgG subclass levels [95% CI] of 157 probands were: IgG1 5.31 g/L [3.04, 9.89]; IgG2 3.56 g/L [1.29, 5.75]; IgG3 0.61 g/L [0.17, 1.40]; and IgG4 0.26 g/L [<0.01, 1.25]. Relative levels of IgG1, IgG2, IgG3, and IgG4 were 54.5%, 36.6%, 6.3%, and 2.7%, respectively (Table 4). We defined IgG subclass levels <2 SD below the corresponding means as subnormal. In the present cohort of 157 hemochromatosis probands, subnormal IgG1, IgG2, IgG3, and IgG4 levels were defined as <3.04 g/L, <1.29 g/L, <0.17 g/L, and <0.01 g/L, respectively.

**Table 4:** Serum IgG subclass levels of adults.

Author (year)	Reference	Adults, n^a^	Method^b^	Mean IgG1, g/L [95% CI] (relative concentration, %)	Mean IgG2, g/L [95% CI] (relative concentration, %)	Mean IgG3, g/L [95% CI] (relative concentration, %)	Mean IgG4, g/L [95% CI] (relative concentration, %)
**Morrell and Skvaril (2023)**	[32]	108	RIA	6.63 (60.9)	3.22 (29.6)	0.58 (5.3)	0.46 (4.2)
**van der Giessen et al. (1975)**	[33]	107	RID (poly)	7.10 (59.4)	3.60 (31.1)	0.65 (5.4)	0.60 (5.0)
**Shakib et al. (1975)**	[34]	111	RID (poly)	8.01 (71.7)	2.17 (19.4)	0.91 (8.1)	0.08 (0.7)
**French and Harrison (1984)**	[35]	172	RID (mono)	5.91 [3.19, 10.2] (60.3)	3.04 [1.23, 6.63] (31.0)	0.61 [0.16, 1.94] (6.2)	0.24 [<0.03, 1.33] (2.4)
**Aucouturier et al. (1985)**	[36]	180	CI-ELISA (mono)	6.35 (65.0)	2.61 (26.2)	0.414 (4.3)	0.456 (4.8%) men; 0.29 (3.2%) women
**Madassery et al. (1988)**	[37]	100	PCFI (mono)	6.1 (61.7)	3.0 (30.3)	0.56 (5.7)	0.23 (2.3)
**Schauer et al. (2003)**	[38]	68	Neph	5.0 [2.8, 8.0] (55.6)	3.0 [1.15, 5.70] (33.4)	0.64 [0.24, 1.25] (6.1)	0.35 [0.05, 1.250] (3.9)
**Harkness et al. (2020)**	[39]	11,673^c^	not reported	5.9 (59.1)	3.1 (31.1)	0.56 (10.9)	0.42 (4.2)
**Present cohort (2023)**	-	157	Neph	5.31 [3.04, 9.89] (54.5)	3.56 [1.29, 5.75] (36.6)	0.61 [0.17, 1.40] (6.3)	0.26 [<0.01, 1.25] (2.7)

^a^Morrel and Skvaril, young adult males; van der Giessen et al., laboratory personnel of known Gm phenotype; Shakib et al., randomly selected normal adults; French and Harrison, healthy blood donors and hospital personnel; Aucouturier et al., Madassery et al., and Schauer et al., healthy blood donors; Harkness et al., Partners Research Patient Data Registry patients >18 y; present cohort, referred adult hemochromatosis probands with *HFE* p.C282Y homozygosity.

^b^RIA, radial immunoassay; RID, radial immunodiffusion; CI-ELISA, competitive indirect enzyme-linked immunosorbent assay; poly, polyclonal antisera against IgG subclasses; mono, monoclonal anti-IgG subclass antibodies; PCFI, particle concentration fluorescence immunoassay; Neph, nephelometry.

^c^ Whites >18 y so identified in a data registry.

Mean and relative levels of IgG subclasses of the present and of eight previously published adult cohorts (n >50) without hemochromatosis diagnoses are displayed in Table 4. Mean IgG1 level of the present cohort was lower than that of seven of the eight other cohorts. Mean IgG2 level of the present cohort was higher than that of seven of the eight other cohorts. Mean IgG3 level of the present cohort was lower than that of three other cohorts. Mean IgG4 level of the present cohort was lower than that of five other cohorts.

Relative IgG1 level of the present cohort was lower than that of eight other cohorts (Table 4). Relative IgG2 level of the present cohort was higher than that of eight other cohorts. Relative IgG3 level of the present cohort was lower than that of two other cohorts. Relative IgG4 level of the present cohort was lower than that five other cohorts.

## Discussion

A novel feature of the present study is characterization of serum IgG subclass levels of a replication cohort of 157 referred adult hemochromatosis probands with *HFE* p.C282Y homozygosity without non-hemochromatosis iron-related disorders or conditions or treatments often associated with abnormal IgG or IgG subclass levels. Median SF, mean BMI, median IgG4, and median phlebotomy units to achieve iron depletion were significantly higher in men than women, typical of adults referred with hemochromatosis [4]. We compared IgG subclass data in the present cohort with those of eight previously published adult cohorts unselected for hemochromatosis diagnoses [32–39].

We observed no significant differences in corresponding IgG1 and IgG2 levels of men and women with hemochromatosis, in agreement with four studies of adults unselected for hemochromatosis diagnoses [35, 36, 38, 39]. Median IgG3 levels of the present men and women did not differ significantly, consistent with a previous report [36], whereas mean IgG3 level of women was 1.2-fold higher than that of men in another study [35]. IgG4 levels were significantly higher in the present men than women. In four other studies, mean IgG4 in men was also significantly higher than that of women [35, 36, 39]. No significant differences between IgG subclass levels of men and women were detected in a study of 68 healthy blood donors [38].

In this study, IgG subclass levels were not significantly associated with TS or phlebotomy units of blood removed to achieve iron depletion. These findings agree with observations in a 2003 cohort of hemochromatosis probands with *HFE* p.C282Y homozygosity [10]. In another study, mean IgG levels of non-pregnant Iranian women with and without iron deficiency did not differ significantly [40]. In Chinese children, mean IgG1 and IgG4 levels were significantly lower in those with than without iron deficiency [41].

IgG1 levels were positively associated with IgG3 levels in this study. Fifteen of the present probands (9.6%) had both IgG1 <4.21 g/L and IgG3 <0.41 g/L at diagnosis. *IGHG1* and *IGHG3* loci, adjacent on chromosome 14q32.33, are in linkage disequilibrium [42]. Antibody responses to soluble and membrane proteins primarily induce IgG1, although lower levels of IgG3 and IgG4 responses to protein antigens also occur [43]. There is a significant positive association of IgG1 and IgG3 levels of adults unselected for hemochromatosis or antibody deficiency diagnoses [44] and subnormal levels of both IgG1 and IgG3 are common in adults with frequent or severe respiratory tract infection [15, 45].

IgG2 levels were positively associated with IgG4 levels in the present study. *IGHG2* and *IGHG4* loci are adjacent on chromosome 14q32.33 [42]. Mean levels of IgG2 and IgG4 are higher in subjects positive rather than negative for the genetic marker Gm(23) [44]. Subnormal levels of IgG2 often occur in association with subnormal IgG4 levels [46].

IgG3 levels were positively associated with IgG1 levels in this study. Subnormal IgG3 levels are often associated with subnormal levels of other IgG subclasses in adults unselected for hemochromatosis diagnoses [31, 45].

IgG1, IgG2, IgG3, and IgG4 levels were not significantly associated with HLA-A*03 positivity or HLA-B*44 positivity, after adjustment for other variables. In Alabama and Portugal hemochromatosis patients with *HFE* p.C282Y homozygosity and positivity for a 500 kb microhaplotype GGG defined by SNPs in chromosome 6p genes *PGBD1*, *ZNF193*, and *ZNF165* [47], IgG3 levels were significantly higher than those of patients homozygous for microhaplotype AAT that is strongly associated with HLA-A*03 [48]. In contrast, IgG1, IgG2, and IgG4 levels did not differ significantly in patients with or without GGG [48]. This suggests that a chromosome 6p locus linked to GGG increases IgG3 levels, consistent with a previous postulate [10]. We found no other report that rs1800562 or *HFE* is associated with IgG subclass levels of humans [49].

In the present cohort, subnormal IgG1, IgG2, IgG3, and IgG4 levels were <3.04 g/L, <1.29 g/L, <0.17 g/L, and <0.01 g/L, respectively. The mean level of IgG1 was lower in the present cohort than in seven of eight previously published cohorts unselected for hemochromatosis [32–39]. The mean level of IgG2 was higher in the present cohort than in seven of eight published cohorts unselected for hemochromatosis [32–39]. These comparisons suggest that IgG subclass levels of referred hemochromatosis probands with *HFE* p.C282Y homozygosity differ from those of adults unselected for hemochromatosis diagnoses. It is plausible although unproven that rates of IgG subclass synthesis or catabolism differ in adults with and without p.C282Y homozygosity. *HFE* RNA expression is low in naive and memory B-lymphocytes and myeloma cell lines [50], although it is unreported whether RNA expression differs in these respective cells with and without p.C282Y/p.C282Y. Differences in study populations, collection, handling, and storage of sera, and methodology, reagents, and instrumentation used to measure IgG subclasses across cohorts we tabulated could also contribute to these differences.

Strengths of this study include evaluation of a large cohort of referred adult hemochromatosis probands with *HFE* p.C282Y homozygosity with HLA-A and -B typing/haplotyping without non-hemochromatosis iron-related disorders or conditions or treatments often associated with abnormal IgG or IgG subclass levels. A limitation of this study is the lack of IgG subclass observations in control cohorts of age- and sex-matched adults with p.C282Y homozygosity identified in population screening and adults with *HFE* wt/wt (lack of p.C282Y and p.H63D (rs1799945)).

The present cohort consisted of non-Hispanic whites. We compared their IgG subclass levels with those in other cohorts that were or presumed to be predominantly white subjects of European descent. Nonetheless, IgG subclass concentrations differ according to race [39]. It is also plausible although unproven that IgG subclass values may differ across non-Hispanic whites of different European ethnicities, nationalities, or derivative countries.

A single clinical laboratory measured all IgG subclasses we report, although it is improbable that the same reagents and instrumentation were used throughout the interval of the present study. Further, it is likely that variables other than those we studied are major determinants of IgG subclass levels. MHC class II typing, chromosome 6p microhaplotyping, detection of IgG subclass alleles, and measurement of specific antibodies and responsiveness to specific antigens were beyond the scope of this study.

## Conclusions

Mean IgG subclass levels of hemochromatosis probands were 5.31, 3.56, 0.61, and 0.26 g/L, respectively. Median IgG4 was higher in men than women. There were positive associations of IgG subclass levels. Mean IgG1 may be lower and IgG2 may be higher in hemochromatosis probands than adults unselected for hemochromatosis.
